# Label-free vibrational imaging of different Aβ plaque types in Alzheimer’s disease reveals sequential events in plaque development

**DOI:** 10.1186/s40478-020-01091-5

**Published:** 2020-12-11

**Authors:** Dominik Röhr, Baayla D. C. Boon, Martin Schuler, Kristin Kremer, Jeroen J. M. Hoozemans, Femke H. Bouwman, Samir F. El-Mashtoly, Andreas Nabers, Frederik Großerueschkamp, Annemieke J. M. Rozemuller, Klaus Gerwert

**Affiliations:** 1 Division of Biospectroscopy, Center for Protein Diagnostics (PRODI), Ruhr University Bochum, Bochum, Germany; 2 Department of Biophysics, Faculty of Biology and Biotechnology, Ruhr University Bochum, Bochum, Germany; 3 Department of Pathology, Amsterdam Neuroscience, Amsterdam UMC - Location VUmc, Amsterdam, The Netherlands; 4 Department of Neurology, Amsterdam Neuroscience, Alzheimer Center Amsterdam, Amsterdam UMC - Location VUmc, Amsterdam, The Netherlands

**Keywords:** Alzheimer’s disease, Amyloid plaque, Human, Amyloid-beta, Oligomer, Fibril, Microspectroscopy, FTIR, Infrared, Raman, Imaging

## Abstract

The neuropathology of Alzheimer’s disease (AD) is characterized by hyperphosphorylated tau neurofibrillary tangles (NFTs) and amyloid-beta (Aβ) plaques. Aβ plaques are hypothesized to follow a development sequence starting with diffuse plaques, which evolve into more compact plaques and finally mature into the classic cored plaque type. A better molecular understanding of Aβ pathology is crucial, as the role of Aβ plaques in AD pathogenesis is under debate. Here, we studied the deposition and fibrillation of Aβ in different plaque types with label-free infrared and Raman imaging. Fourier-transform infrared (FTIR) and Raman imaging was performed on native snap-frozen brain tissue sections from AD cases and non-demented control cases. Subsequently, the scanned tissue was stained against Aβ and annotated for the different plaque types by an AD neuropathology expert. In total, 160 plaques (68 diffuse, 32 compact, and 60 classic cored plaques) were imaged with FTIR and the results of selected plaques were verified with Raman imaging. In diffuse plaques, we detect evidence of short antiparallel β-sheets, suggesting the presence of Aβ oligomers. Aβ fibrillation significantly increases alongside the proposed plaque development sequence. In classic cored plaques, we spatially resolve cores containing predominantly large parallel β-sheets, indicating Aβ fibrils. Combining label-free vibrational imaging and immunohistochemistry on brain tissue samples of AD and non-demented cases provides novel insight into the spatial distribution of the Aβ conformations in different plaque types. This way, we reconstruct the development process of Aβ plaques in human brain tissue, provide insight into Aβ fibrillation in the brain, and support the plaque development hypothesis.

**Electronic supplementary material:**

The online version of this article (10.1186/s40478-020-01091-5) contains supplementary material, which is available to authorized users.

## Introduction

Alzheimer’s disease (AD) is the most common neurodegenerative disease and is pathologically characterized by hyperphosphorylated tau neurofibrillary tangles (NFT) and amyloid-beta (Aβ) plaques. Aβ originates from the cleavage of the amyloid precursor protein (APP) and is secreted to the extracellular space. The most accepted hypothesis for AD pathogenesis is the amyloid cascade hypothesis [32, 66]. According to this hypothesis, Aβ aggregates in the neuropil as plaques, due to an imbalance of Aβ production and clearance. The Aβ monomers misfold and form β-sheet-rich oligomers, which then form protofibrils that stack into highly organized amyloid fibrils [38, 50]. The aggregation of Aβ causes synaptic stress and induces an inflammatory response. Simultaneously, synaptic and neuronal injury leads to the hyperphosphorylation of tau, which aggregates within neurons as NFTs that finally cause neuronal death. As the disease spreads and progresses, there is extensive neuronal death throughout the brain, which ultimately leads to dementia. The amyloid cascade hypothesis is currently under debate. While it is proposed that Aβ is the initial trigger of pathological processes, NFTs are considered to be the progressive force of the disease [55]. The discussion is fueled by several failed clinical studies of Aβ-targeting antibodies, as well as encouraging results of most recent anti-Aβ drug studies [77, 12, 67].

Aβ plaques show different morphologies. Here, we consider (i) the diffuse type, (ii) the compact (or primitive) type, and (iii) the classic cored type [76]. It is proposed that these different morphologies represent the progressive stages of Aβ fibrillation [7, 34, 62, 75]. Plaque formation is proposed to start as diffuse amorphous structures that mainly consist of aggregated Aβ oligomers and protofibrils. Then, with the progression of Aβ fibrillation, the plaque shows an increasingly compact morphology with a more clearly defined outline. An inflammatory response, driven mainly by microglia, is strongly associated with the early stages of Aβ plaque formation and even considered to drive the continuing build-up of amyloid fibrils and the accompanied neurotoxic effects [48, 61, 74]. The final fibrillation stage is reached when Aβ is condensed to a core that contains mostly Aβ fibrils.

Here, we applied Fourier transform infrared (FTIR) and Raman imaging to snap-frozen thin sections of human brain tissue. These label-free methods are much less invasive towards the sample than staining methods because the tissue is examined without chemical alterations [30]. The vibrational microspectroscopy approach provides spatially resolved spectra that reflect the biochemical fingerprint of analyzed samples, including the protein secondary structure [21, 26, 31]. Raman is a complementary spectroscopic technique to FTIR and is used here to verify the FTIR results. The major constituents of brain tissue are proteins and lipids [53]. The secondary structure of proteins can be determined by analyzing the Amide I absorbance band (C=O stretching vibration of the protein backbone). The Amide I absorbance band is indicative for the secondary structure. It consist of several bands, each associated with distinct secondary structures [28, 29, 37]. The position of the main β-sheet-band around 1630 cm^−1^ shifts towards lower wavenumbers, when the strands become arranged in parallel β-sheets [9, 47, 78]. Accordingly, amyloid fibrils often absorb at a lower wavenumber than native β-sheet proteins [83]. For instance, oligomeric Aβ with typically antiparallel β-sheet structure absorbs around 1630 cm, whereas a shift to lower wavenumbers has been reported for Aß fibrils [5, 41, 54, 63, 64]. Furthermore, antiparallel β-sheets display a characteristic band around 1693 cm^−1^. This band is n^−1^ot observed in Aβ fibrils with predominantly parallel β-sheets [8, 10]. Here, the accumulation of β-sheet-rich Aβ oligomers and fibrils in plaques is studied, analyzing the Amide I band. Apart from that, lipids constitute about 40% of the gray matter dry weight [56] and show characteristic absorbance bands as well. The fatty acids in lipids consist mostly of methylene and methyl (CH_2_ and CH_3_ groups, which also occur in protein side chains) that generate stretching vibration bands in the region 3000–2800 cm^−1^. The head groups of most phospholipids, which make up ~ 70% of the lipid content, contain ester groups that generate the lipid-associated band (ester C=O stretching vibration) around 1738 cm^−1^ [18, 43].

In this study, the progression of Aβ fibrillation, alongside the proposed development sequence of Aβ plaques (diffuse, compact, classic cored) in AD is studied with spatial and molecular resolution, using label-free imaging. Post-mortem sections from fresh-frozen brain tissue were analyzed by FTIR and Raman imaging without chemical tissue treatment to stay as close to the brain’s conditions as possible. Particularly, the secondary structure-sensitive Amide I band was analyzed spatially resolved in different Aβ plaque types. Plaques in the analyzed region were subsequently confirmed by anti-Aβ immunohistochemistry (Aβ-IHC) on the same tissue section. We observed increased Aβ fibril contents alongside the ascending plaque stages. The spectral image analysis provides insight into the spatial distribution of Aβ structure in different plaque types, contributing evidence for the current hypotheses on plaque development.

## Materials and methods

### Post-mortem human brain tissue

Post-mortem brain tissue was selected from the Netherlands Brain Bank (the NBB; Amsterdam, The Netherlands). Donors or their next of kin signed informed consent for the usage of brain tissue and clinical information for research purposes. The Institutional Review Board and Medical Ethical Board from the Vrije University Medical Center approved the procedures of the NBB. Neuropathological diagnosis was performed (by A.J.M.R) and was based on multiple (immuno)histochemical stainings of diversified brain regions according to the standard operating procedures of the NBB and BrainNet Europe consortium. AD cases (n = 5) were selected when clinical and neuropathological information fulfilled the criteria of the National Institute on Aging-Alzheimer’s Association (NIA-AA) for AD and no other neurodegenerative or psychiatric disease was present [51]. Two additional AD cases with the vascular type were included—also referred to as CAA-Type 1. These cases do not fit the typical NIA-AA criteria, since amyloid and tau depositions are vascular related. Controls were selected when no cognitive decline was reported during life and AD pathology was absent or ‘low’ (Additional file 1: Table 1). Snap-frozen tissue of the superior parietal lobule (LPS) was used, as this neocortical area shows the plaque types of interest. Sections (20 µm) were mounted on CaF_2_ slides for vibrational imaging and subsequent Aβ-IHC. Tissue sections were stored at − 80 °C in-between experiments in order to minimize sample degradation [46].

### FTIR imaging

FTIR was conducted with a *Cary 670* spectrometer (Agilent Technologies), coupled to a *Cary 620* microscope (Agilent Technologies) in transmission mode. The microscope features a 128 × 128-element focal plane array detector and a 15 × (0.62 NA) objective. In high magnification mode (5 × optical increase), the instrument yields a nominal pixel size of 1.1 µm. Each 128 × 128-element data acquisition provided a field of view (FOV) of approximately 141 × 141 µm. interferograms were obtained as a mean of 128 scans. Using *Blackman-Harris*-4-term apodisation, power phase correction and zero-filling factor 2 for Fourier-transformation, the resulting spectral range was 3700–948 cm^−1^ at a spectral sampling interval of 1.9 cm^−1^. For background correction, a clean area of each CaF_2_ slide was measured (1024 scans) and subsequently subtracted from sample measurements. The software *Resolutions Pro 5.3* was used for image acquisition. The instruments and the sample cavity were continuously purged with dry air to reduce atmospheric water vapor contribution and to maintain the samples in a conserving dry state. We checked for spectral alterations that may have been caused by prolonged exposure to the dry air environment during the experiments. None of the bands used here for plaque analysis showed noteworthy alterations (Additional file 1: Fig. 1).

### Immunohistochemical staining

Following spectral measurements, the sample was placed into a container filled with argon at a temperature of 36 °C for one hour to increase the tissue adherence whilst maintaining dry conditions. The sample was fixated in ethanol for 10 min, and dipped in a gelatin solution (0.3% in 50 mM Tris–HCL buffer) to further increase tissue adherence. After washing (3 × 5 min in PBS (Thermo Fisher)), the sample was incubated with the primary antibody mouse-anti-Aβ directed against aa1-16 (clone IC16) in antibody diluent (*Agilent Dako*) for one hour. After washing, the sample was incubated with *Envision* (*Agilent Dako*) for 1 h and washed. Color development was done using *DAB* (*Agilent Dako*). The section was dehydrated in an ethanol series (70–96–100%), mounted with Euporal (Roth) and coverslipped. The stained sample was subsequently imaged with an *Olympus BX61VS* slide scanner, using the *UPlanSApo 20* × *0.75 NA* objective (Olympus). The exemplary classic cored plaque appears to have a hollow core in the Aβ-IHC image (Fig. 2A_4_). This phenomenon was described before [65] and may derive from incomplete antibody penetration into the plaque core during the Aβ-IHC staining procedure. Another classic cored plaque with a homogeneously stained core exhibits similar distributions of (β-sheet) protein (Additional file 1: Fig. 2).

### Image alignment

**Fig. 1:**
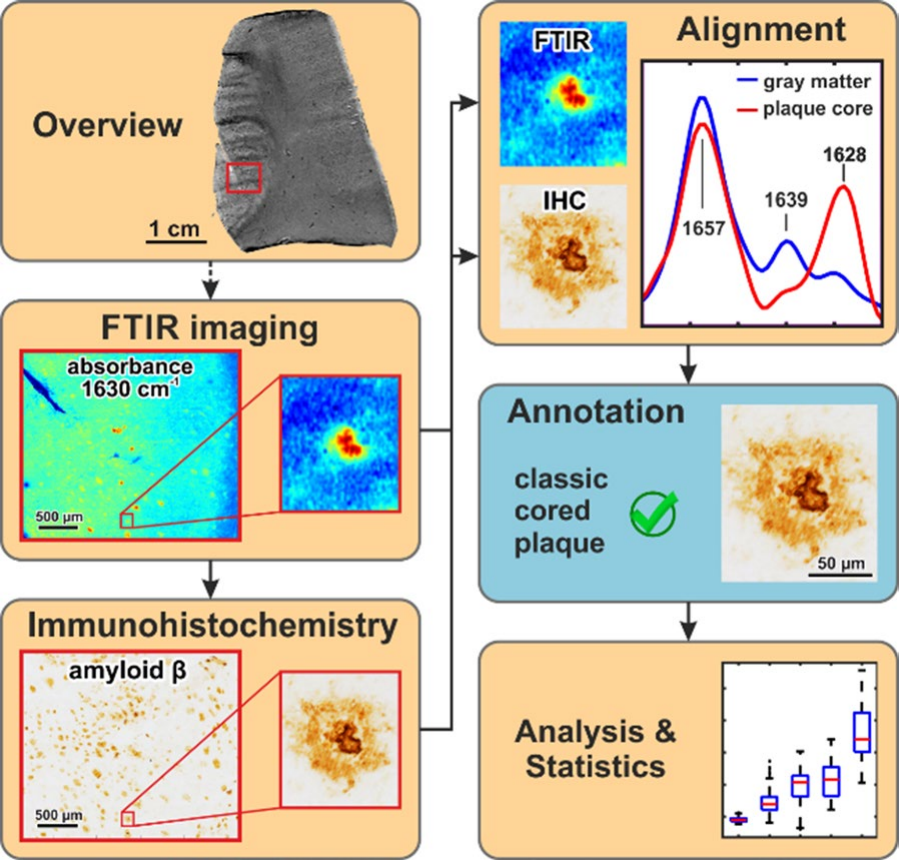
Workflow. Fourier-transform infrared (FTIR) and Raman imaging were applied to selected sample areas. Subsequently, the sample was immunostained against amyloid beta (Aβ) and imaged with light microscopy. The resulting (spectral) images were spatially aligned to generate a precisely overlaid, unified dataset. An experienced neuropathologist (B.D.C.B) annotated Aβ-IHC images to the different plaque types. Based on this data, spectral analysis was conducted and statistically evaluated

In order to link spectral data with Aβ-IHC images, both modalities were precisely overlaid. We used a homemade software (written in *Matlab*) to determine an affine 2D transformation that warps the Aβ-IHC image onto the coordinate system of the vibrational images, based on reference coordinates given by the user. The quality of the overlay was visually verified, based on tissue morphology. Subsequently, a region of interest (ROI) within the spectral coordinates was chosen for each plaque. The ROI of the spectral image and the corresponding region of the Aβ-IHC image were cut out and saved for further analysis. Exemplary cutouts are shown in Fig. 1.

### Plaque annotation

The cutouts of the Aβ-IHC image, containing one plaque each, were transferred to an AD neuropathology expert (B.D.C.B) who has extensive experience with plaque morphologies. No further information was given; the expert was blinded regarding case numbers and disease stages. The expert assigned each plaque to one of the following classes: (1) diffuse plaque, (2) compact plaque, (3) classic cored plaque, (4) miscellaneous, (5) no assignment possible. In this study, only plaques of the diffuse, compact and classic cored type were used.

### FTIR spectral data preparation

Spectra, which were not suitable for analysis, were determined by their noise level (SNR < 100). In addition, spectra with strong scattering contribution (A_silent_ _region_ > A_1655_/2) were excluded. Scattering appears as apparent absorbance in the so-called “*silent region*” (e.g. 2300–1800 cm^−1^), where biological samples usually display no absorbance (Additional file 1: Fig. 3). Thus, a small fraction of spectra was excluded from analysis in order to prevent statistical distortions by outlier spectra. All remaining spectra were subjected to Mie scattering correction, based on extended multiplicative signal correction (EMSC) [42, 70]. Additional file 1: Fig. 3 presents exemplary *plaque spectra* before and after the application of EMSC.

In order to identify spectra within a hypercuboid that correspond to a plaque (more precisely “Aβ positive area”); a binary mask was generated from the Aβ-IHC image cutouts (Additional file 1: Fig. 4), using Otsu’s method (*Matlab Image Processing Toolbox)* [57]. The henceforth-called *plaque spectrum* is the arithmetic mean of all pixel-spectra within this mask. Another mask was generated that describes a ring-shape with a radial thickness of 100 µm, surrounding the plaque, excluding Aβ-positive pixel, which were again detected by using Otsu’s method. The arithmetic mean of the spectra corresponding to the latter mask is henceforth called *surrounding spectrum*.

### FTIR spectral data analysis

For the structural analysis of each classic cored plaque, the respective spectral image cutout was separated using hierarchical cluster analysis (HCA) (Matlab, Statistics toolbox). Spectra were thereby grouped into 10 subgroups based on spectral similarity. The subgroups belonging to the core or the corona were each selected by visual comparison to the corresponding Aβ-IHC image and merged, if applicable. The arithmetic means of spectra belonging to the core were used in further analysis and are being referred to as *core spectra* of the *inner core*. Difference spectra were calculated between *plaque spectra* and *surrounding spectra*. For the analysis of the Amide region, a linear baseline was subtracted from the spectra within the range 1800–1480 cm^−1^. Whereas, for the detailed investigation of the band around 1630 cm^−1^, a linear baseline was subtracted from the Amide I bands within the range 1690–1610 cm^−1^. The bands were subsequently area-normalized in the range 1690–1610 cm^−1^ and subtracted from each other. Arithmetic means of all difference spectra from a respective plaque-type were calculated and interpolated with a spline for display. Second derivatives of spectra were calculated, using a *Savitzki-Golay* filter with a third-degree polynomial and a frame size of five (Matlab, *Signal Processing toolbox*).

### FTIR band assignment and interpretation

We determined spectroscopic ratios between FTIR absorbance bands that correspond to vibrational modes of the protein backbone, CH_2_, and CH_3_ groups (Additional file 1: Table 2). The protein accumulation is quantified by the Amide II peak (A_1545_, N–H bending vibration) of the protein backbone vibration [45], in proportion to the absorbance in the CH-stretching region, as proxy for total absorbance from lipids and proteins. The β-sheet levels are quantified by the ratio A_1630 _/A_1655_ of respective bands representing β-sheets and non-β-sheet secondary structures. The band positions were derived from second derivatives. The band around 1655 cm^−1^ is commonly associated with α-helical proteins, but as e.g. unordered structures may also contribute to the band, we use the term *non-β-sheets* here.

### Statistical analysis

The spectroscopic ratios and band height differences in second derivatives were used for statistical analysis. Additional to the Aβ plaques, 208 randomly selected small (approx. 100 × 100 µm), Aβ-negative areas from the gray matter of  non-demented cases constituted the control group. Furthermore, *core spectra* were included as a separate group. In between each group, p-values were calculated alongside the proposed development sequence (Matlab, Statistics toolbox), according to Student’s t-test [71]. The confidence levels were determined according to p-values < 0.05 (*), < 0.01 (**) and < 0.001 (***). The correlation between the ratios was quantified by the Pearson correlation coefficient R [44] (Matlab, Statistics toolbox), using only *plaques spectra* and *core spectra*. We investigated the statistical influence of cases, which contributed high amounts of plaques to the respective types (cases 7 and 9, Additional file 1: Table 1). We found that case 7 has little impact on the statistical analysis of diffuse plaques, whereas the classic cored plaques of case 9 shift the respective distributions slightly downwards, by approx. − 0.16 points for protein and approx. − 0.09 points for β-sheets. Further, we found no notable alterations in the spectral data of case 7, which had the longest post-mortem interval (10.45 h).

## Results

We developed a workflow that combines FTIR, Raman, and Aβ-IHC imaging within the same tissue thin section, thereby integrating label-free imaging with the neuropathology gold standard for plaque detection (Fig. 1). In each sample, areas of about 20 mm^2^ were measured by FTIR imaging. Additionally, smaller subareas of about 0.2 mm^2^ were imaged by Raman. Subsequently, the same tissue section was immunostained against Aβ and imaged by light microscopy. By spatial overlay of vibrational and Aβ-IHC images, Aβ plaques were clear-cut identified in FTIR and Raman images. AD neuropathology experts annotated each scanned plaque in Aβ-IHC images to differentiate between the plaque types. We present the spectral analysis of 68 diffuse plaques [80], 32 compact plaques [16], and 60 classic cored plaques [69], measured in 20 µm thick native post-mortem brain sections of 7 AD cases. The plaques were compared to their surrounding tissue (Additional file 1: Fig. 4) and Aβ-negative gray matter of 3 non-demented control cases (Additional file 1: Table 1). None of the bands used here for analysis showed alterations during the measurements (Additional file 1: Fig. 1), ruling out sample degradation.

### FTIR imaging reveals the distribution of ß-sheet protein in different plaque types

**Fig. 2:**
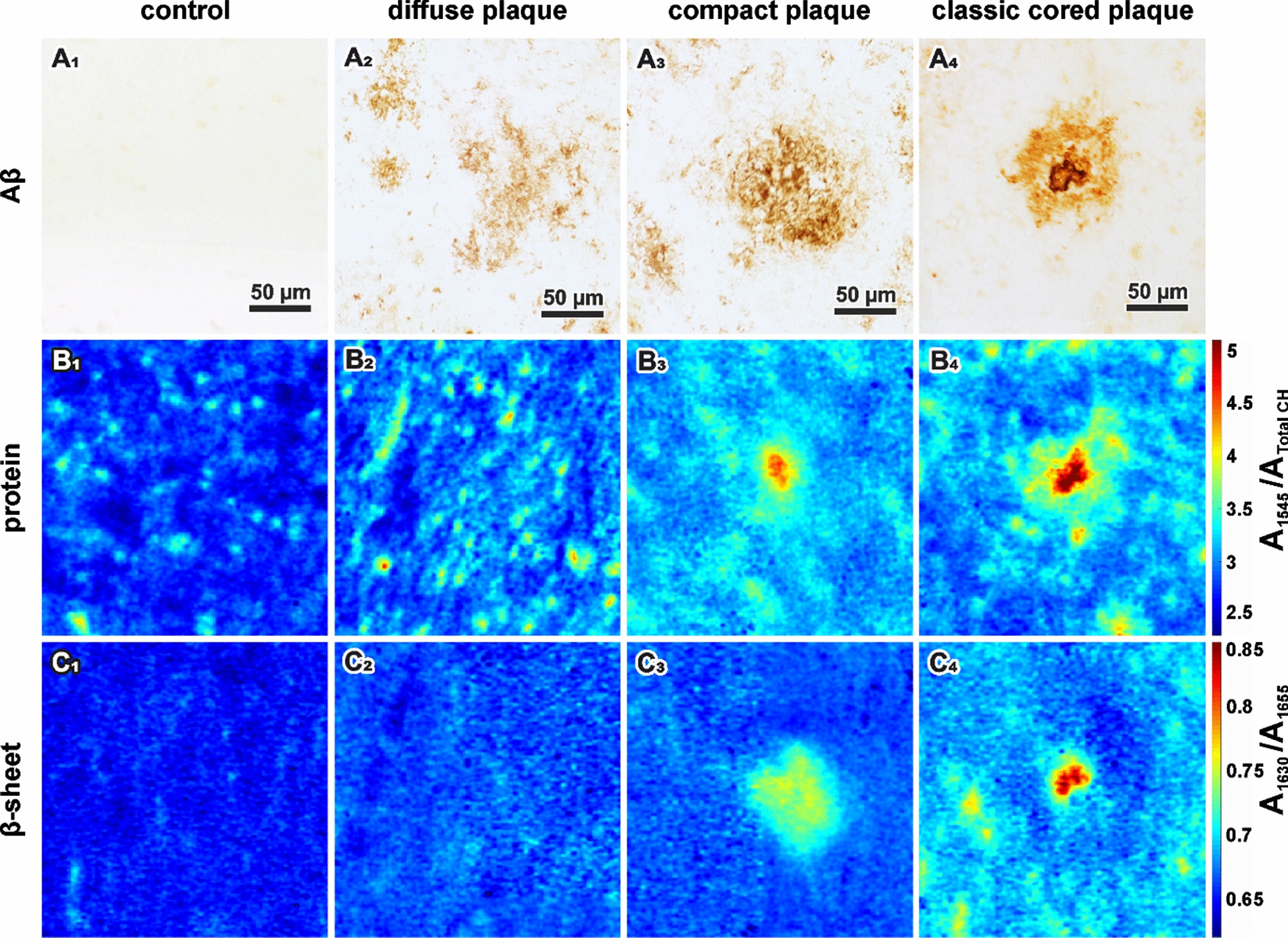
Immunohistochemical and FTIR imaging of control tissue (_1_) diffuse (_2_), compact (_3_) and classic cored plaques (_4_). **A** Immunohistochemical staining against amyloid beta (Aβ). **B** Ratio between the Amide II and CH stretching bands. Red indicates high protein concentrations. **C** Ration between the main β-sheet band and non-β-sheet band of the Amide I. Red indicates high β-sheet levels

We present exemplary plaques of each type in Aβ-IHC images (Fig. 2A). Healthy control tissue is shown as a reference (Fig. 2A_1_). The FTIR results of the same tissue areas are visualized in pseudo-color images that display protein accumulation (Fig. 2B). We observed increased protein concentrations in all plaque types, compared to control tissue. We assign this aggregated protein mostly to Aβ, as indicated by the respective Aβ-IHC images (Fig. 2A). Due to the cellular composition of brain tissue, the protein concentration in control tissue is not homogeneous (Fig. 2B_1_). The diffuse plaque shows protein accumulations that are randomly distributed within the plaque area (Fig. 2B_2_). In the compact plaque, protein is accumulated in the center (Fig. 2B_3_). Overall, compact plaques displayed a wide range of protein distributions, spanning from homogeneous dispersions to centralized accumulations. Interestingly, in some compact plaques we even noted core-like protein structures (Fig. 2B_3_), which were not visible in corresponding Aβ-IHC images (Fig. 2A_3_). Classic cored plaques, recognized by their typical core in Aβ-IHC images (Fig. 2A_4_), present in FTIR data a core with high protein content that is surrounded by a corona with less aggregated protein (Fig. 2B_4_).

In the next step, we analyzed the secondary structure composition of Aβ in the different plaque types to evaluate β-sheet levels (Fig. 2C). This is elaborated from the ratio between the Amide I bands of β-sheets and non-β-sheets. Healthy gray matter shows no β-sheet aggregations (Fig. 2C_1_). In diffuse plaques, we observed slightly increased β-sheet levels (Fig. 2C_2_). The compact plaque shows increased β-sheet levels across the plaque area (Fig. 2C_3_). The classic cored plaque shows aggregated β-sheet protein, condensed in its core (Fig. 2C_4_). Interestingly, the surrounding corona displays low levels of β-sheets, similar to diffuse deposits. Two selected classic cored plaques were additionally analyzed with Raman imaging, confirming FTIR observations (Additional file 1: Fig. 6).

**Fig. 3:**
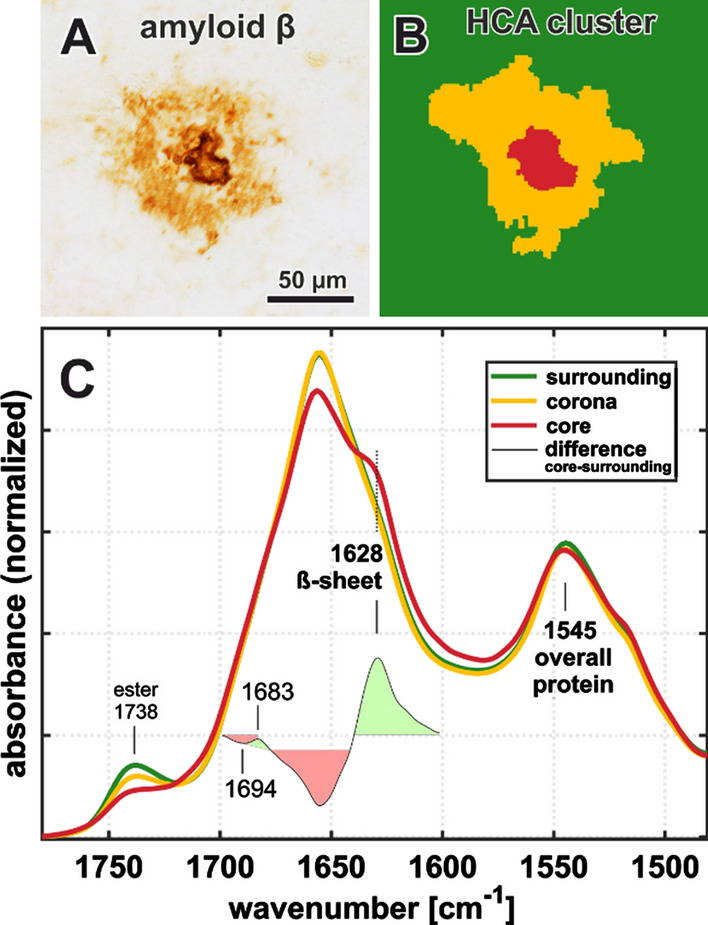
FTIR analysis of an exemplary classic cored plaque and its compartments. **A** Anti-Aβ immunostaining **B** Areas of spectral similarity, identified by hierarchical cluster analysis (HCA), that correspond to surrounding tissue (green), corona (yellow) and core (red). **C** Area-normalized FTIR spectra in the range 1780–1480 cm^−1^. The red core spectrum shows a prominent shoulder around 1628 cm^−1^, and reduced absorbance around 1655 cm^−1^. A difference spectrum (black) in the in the range 1700–1600 cm^−1^ reveals minor bands around 1683 cm^−1^ and 1694 cm^−1^. The lipid-associated ester band around 1738 cm^−1^ is decreased in both the corona and the core

The characteristic core and corona structure of classic cored plaques was analyzed in more detail (Fig. 3). Plaque cores and coronas were distinguished from each other and from the surrounding tissue, based on their spectral properties, using hierarchical cluster analysis (HCA). The method is described above. We performed HCA on spectral hypercuboids of all included classic cored plaques (n = 60). Thereby, we separated pixel that correspond to the core of each plaque and generated *core spectra* of 52 out of the 60 classic cored plaques. The remaining cores were either too small to be resolved in FTIR or were discarded due to insufficient data quality. HCA results for the exemplary classic cored plaque are shown (Fig. 3A, B). Figure 3C shows only the most relevant spectral range, containing the Amide I (~ 1655 cm^−1^), Amide II (~ 1545 cm^−1^), and the lipid-associated ester band (~ 1738 cm^−1^). The ester band is decreased in the corona (yellow) and even more in the core (red). However, we note that the ester band does not disappear entirely, indicating a residual lipid content within the core. The structure-sensitive Amide I band of the corona is nearly identical to that of the surrounding tissue (green), indicating low β-sheet levels in the corona. In contrast, the *core spectrum* displays a strong shoulder around 1628 cm^−1^. A difference-spectrum between the core and the surrounding tissue is shown (Fig. 3C). It reveals positive bands around 1683 cm^−1^ and 1628 cm^−1^, as well as negative bands around 1694 cm^−1^ and 1655 cm^−1^.

### The Amide I band reveals structural properties of the Aβ in plaques

**Fig. 4:**
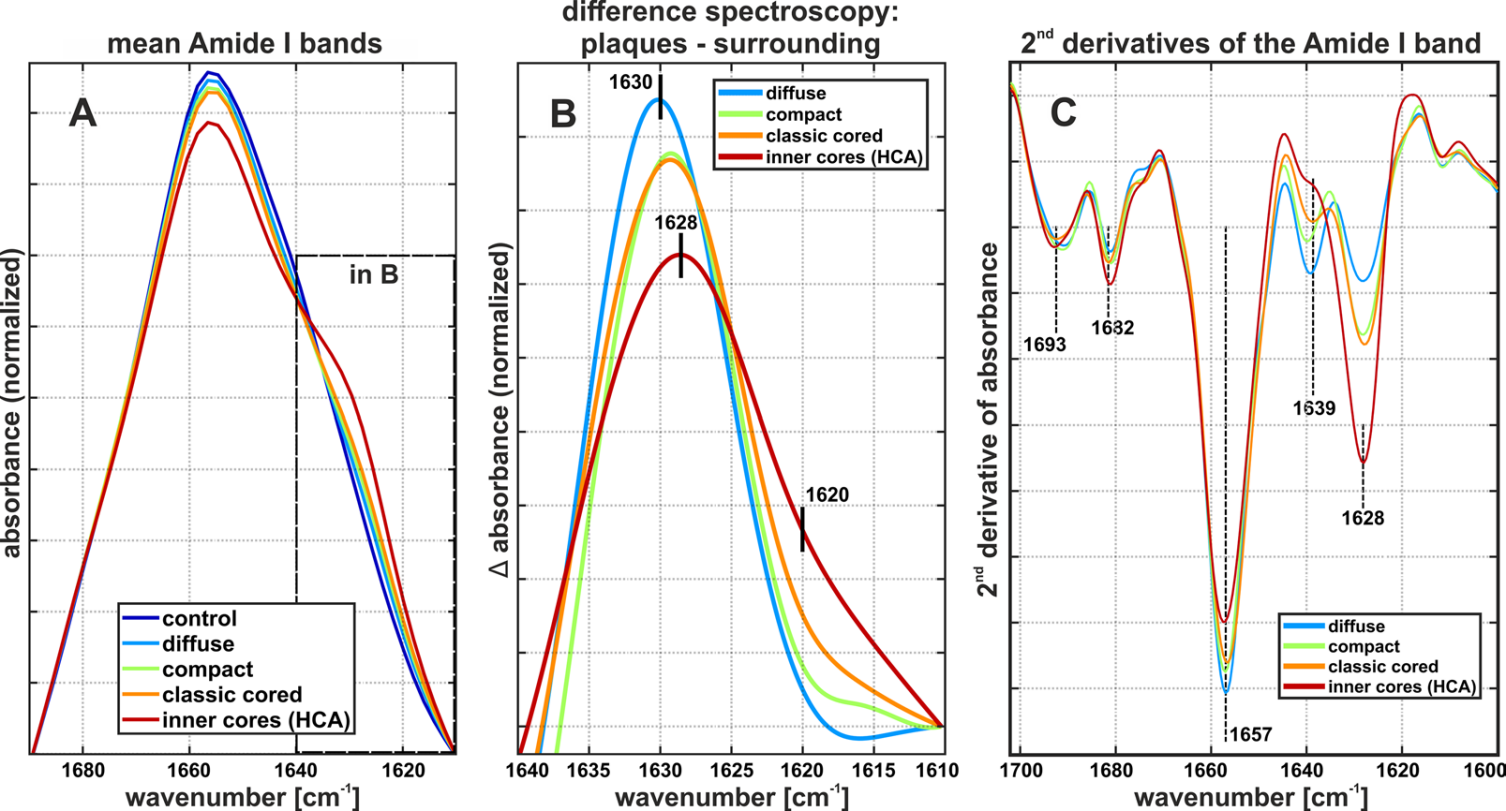
Amide I band analysis. **A** Mean Amide I bands of all plaque spectra from each plaque type, core spectra generated by hierarchial cluster analysis (HCA), and gray matter control spectra. The shoulder around 1628 cm^−1^ indicates β-sheet protein. **B** Cutout of mean difference spectra between plaque spectra and their respective surrounding spectra. Note the shift to lower wavenumbers and the increased absorbance around 1620 cm^−1^. **C** Visualization of sub-bands of the Amide I in the region 1700–1600 cm^−1^. The marked local minima indicate bands that are relevant for protein secondary structure. Note the substantial increase of the band around 1628 cm^−1^ alongside the plaque development sequence. The band around 1693 cm^−1^ displays little change, whereas the band around 1682 cm^−1^ increases, and the bands around 1657 cm^−1^, and 1639 cm^−1^ decrease

A detailed analysis of the Amide I band reveals further differences between the plaque types (Fig. 4). The previously described, increased absorbance around 1630 cm^−1^ manifests in a shoulder on the right side of the Amide I band (Fig. 4A). Difference spectra between *plaque spectra* and *surrounding spectra* reveal that the 1630 cm^−1^ band shifts to the right, alongside the proposed plaque development sequence (diffuse, compact, classic cored) (Fig. 4B). The band position reaches 1628 cm^−1^ in *core spectra*. Additionally, the right flank of the band broadens, showing increased absorbance around 1620 cm^−1^. The shift to lower wavenumbers is reproduced in second derivative spectra (Fig. 4C), which reveal additional bands around 1693 cm^−1^, 1682 cm^−1^, 1657 cm^−1^, and 1639 cm^−1^. The band around 1682 cm^−1^ is slightly increased, whereas the band around 1693 cm^−1^ does not change and the bands around 1657 cm^−1^, and 1639 cm^−1^ are decreased. In accordance, we observe a positive band around 1683 cm^−1^ and a negative band around 1694 cm^−1^ emerging in difference spectra (Additional file 1: Fig. 5).

**Fig. 5:**
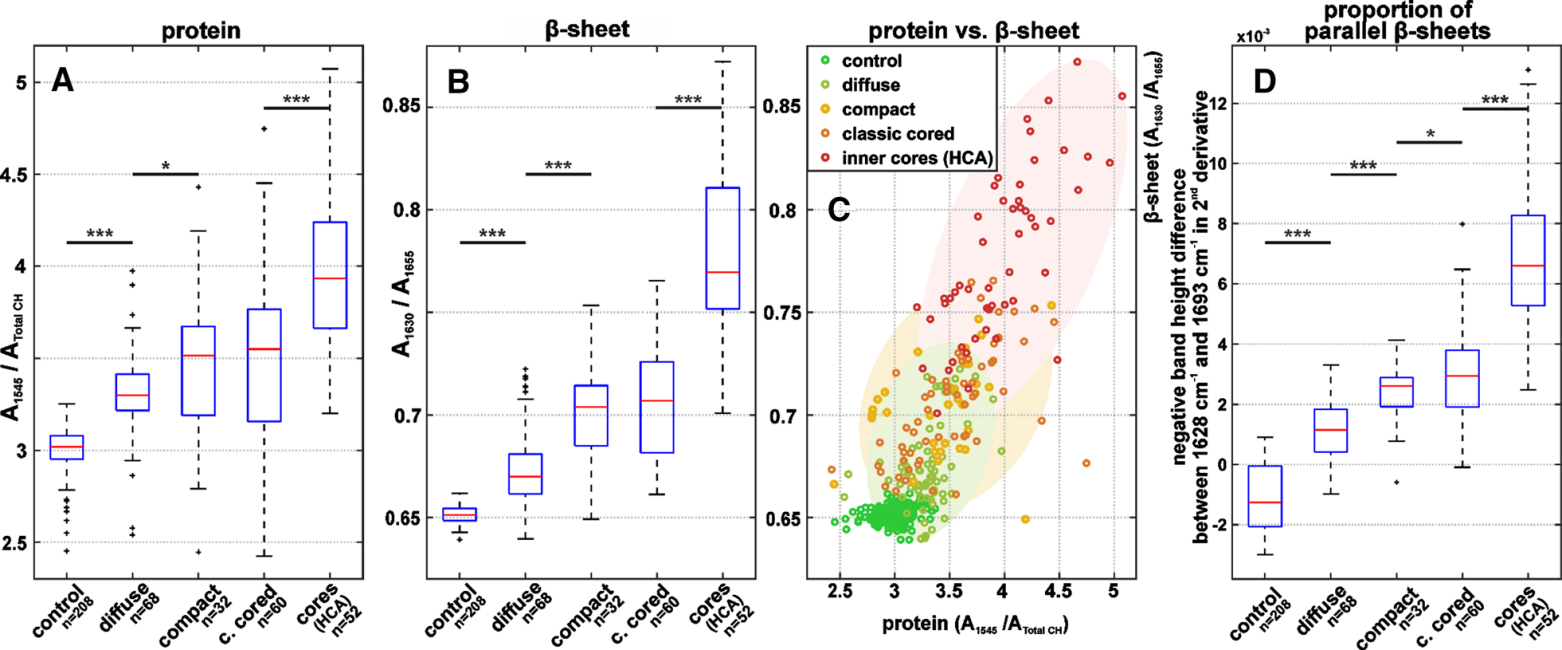
Statistical analysis. The boxplots present spectroscopic ratios derived from *control,* *plaque*, and *core* *spectra*. The red bar indicates the median value, the blue boxes range between the first and third quartile. The black whiskers extend the extremes of the distribution, excluding outliers (black crosses). The significance bars announce the confidence levels. **A** Ratios between the Amide II band and the CH stretching bands, indicating protein accumulation. **B** Ratios between the Amide I band of β-sheets and non-β-sheet structures, indicating β-sheet levels. **C** The scatterplot illustrates the correlation between protein and β-sheet levels in plaques. A successive accumulation of β-sheet protein alongside the plaque development sequence is apparent. **D** The negative height difference of the bands around 1628 and 1693 cm^−1^ in 2^nd^ derivative spectra, indicating increased proportions of parallel β-sheets

Since the biochemical composition of the gray matter is inhomogeneous, especially during disease processes, it is essential to include a viable amount of plaques from a representative cohort for statistical analysis (Fig. 5). The accumulation of protein, as well as the level of β-sheets increase along the sequence from (i) gray matter control, to (ii) diffuse plaques, (iii) compact plaques, and peak in the (vi) cores of classic cored plaques (Fig. 5A, B). The β-sheet levels in diffuse plaques display some outliers (black crosses in Fig. 5B). We associate these outliers with unusually high misfolding levels with the two oldest cases in this study (cases 5 and 6, Additional file 1: Table 1). Further research will be necessary to elaborate if there is a correlation between age and β-sheet levels in diffuse plaques. Notably, we detect no significant differences between *plaque spectra* of compact plaques and classic cored plaques, neither regarding protein nor β-sheet levels. Remarkably, protein and β-sheet levels correlate (R = 0.73) in plaques (Fig. 5C). Furthermore, the height difference between the bands around 1628 cm^−1^ and 1693 cm^−1^ increases significantly alongside the plaque development sequence (Fig. 5D), indicating an increasing dominance of the 1628 cm^−1^ band over the 1693 cm^−1^ band in mature plaques.

## Discussion

We observed the accumulation of Aβ with β-sheet structure in plaques, which centralizes successively alongside the proposed plaque development sequence (Fig. 2). With increasing Aβ density, *plaque spectra* show a shift of the band around 1630 cm^−1^ towards lower wavenumbers and a decreasing band around 1693 cm^−1^ (Figs. 3, 4). This behavior is consistent across cases and shows a steady transition along the proposed plaque development sequence (Fig. 5).

Several studies have already applied vibrational imaging to analyze amyloid brain deposits and thereby provided insight into protein misfolding in tissue. The tissue sections were usually not immunostained against Aβ, as the staining of brain tissue on non-adhesive crystal slides is difficult. Therefore, a differential analysis of Aβ plaque types has not been performed yet. We have overcome this challenge with an optimized staining protocol (see methods) and now present a detailed analysis of the most common Aβ plaque types. Most previous studies used mouse models of AD, as the tissue of transgenic mice is more easily available than human tissue and usually contains dense amyloid deposits [24, 35, 41, 45, 58, 59, 72, 73]. Much has been elaborated about the properties of plaques this way. Surowka et al. report increased β-sheet levels in mature plaques in mice, using FTIR [73]. Fonseca et al. detect lipid-rich cell-sized depositions surrounding mature plaques using Raman microspectroscopy [24].

However, as the transferability of findings between mouse models and humans remains unclear, we investigate Aβ plaques in human brain tissue here, in order to resemble the disease course and pathology in human brains. This is particularly relevant for the interplay between Aβ and the various surrounding cell types, which is considered to be crucial in plaque development [2, 60, 62, 79, 82]. Several groups have applied vibrational imaging to human AD brain tissue before [1, 6, 22, 23, 39, 46, 49]. For instance, Benseny-Cases et al. report an increased 1630 cm^−1^ to 1650 cm^−1^ ratio in Thioflavin T-positive deposits from the human brain [6], which aligns well with our results on classic cored plaques (Fig. 2C_4_). Michael et al. report a shift of the Raman Amide I band towards 1666 cm^−1^ in amyloid deposits [49], which aligns with our observations in plaque cores (Additional file 1: Fig. 6). Klementieva et al. recently detected increased β-sheet levels in neuron models of AD, using second derivatives of optical photothermal infrared spectra [40], which reveal a structure of the Amide I band that is similar to the here presented data (Fig. 4C). Some of the spectroscopic studies on amyloid deposits in formaldehyde-fixated human brain tissue reported elevated lipid bands in ring-like shapes around amyloid cores, which were proposed to originate from microglia [6, 39, 49]. As we do not observe similar structures in our fresh-frozen samples, we suspect that the described lipid-like signal might be caused by the fixation procedure with formaldehyde, which is known to bind to proteins and shows lipid-like absorbance [11]. Two studies report FTIR measurements on amyloid deposits in native human brain tissue [23, 46]. The therein-presented spectra show visibly increased absorbance around 1630 cm^−1^ and decreased absorbance around 1738 cm^−1^, which is in nice agreement with our results (Fig. 3C).

By analyzing the Amide I band in plaques, we conclude a successive accumulation of Aβ with β-sheet structure from increased absorbance around 1630 cm^−1^ (Figs. 2, 5A, B) alongside the proposed plaque development sequence (diffuse, compact, classic cored). Additionally, we observe an increased peak around 1683 cm^−1^ (Fig. 3C and 4C) that is associated with β-turns in β-sheet proteins [27], confirming the accumulation of β-sheet protein. In contrast, we do not observe increased absorbance around 1693 cm^−1^ (Fig. 4C), which would be expected if the deposited Aβ were in an antiparallel β-sheet formation [8, 10]. On the contrary, difference spectra reveal a decreased contribution of the 1693 cm^−1^ band in compact and classic cored plaques (Fig. 3C and Additional file 1: Fig. 5). We deduce that Aβ adopts a parallel β-sheets structure during plaque development [8, 9]. Further, we observe a shift of the Amide I band from around 1630 cm^−1^ to 1628 cm^−1^, accompanied by increased absorbance around 1620 cm^−1^ (Fig. 4B). This shift is associated with a growing number of strands in parallel β-sheets [9, 47, 78]. We conclude that increasing fractions of Aβ is in extended parallel β-sheet conformation (Figs. 4, 5D). Such a shift was described for the formation of Aβ fibrils [5, 54, 41, 63]. The absorbance of amyloid fibrils at low wavenumbers was described in vitro [3, 81, 83] as well as for Aβ fibrils in cell culture [4, 41]. In summary, we conclude the growth of parallel β-sheet fibrils alongside the plaque development sequence, in agreement with other reports [79, 82]. The extensive knowledge about Aβ fibrillation from spectroscopic studies [5, 41, 54, 63, 64] allows us this careful analysis of Aβ in the brain, with respect to the limitations set by the complex conditions in tissue. Thus, to the best of our knowledge, we report the first evidence of Aβ fibril growth alongside the proposed plaque development sequence in the human brain.

**Fig. 6:**
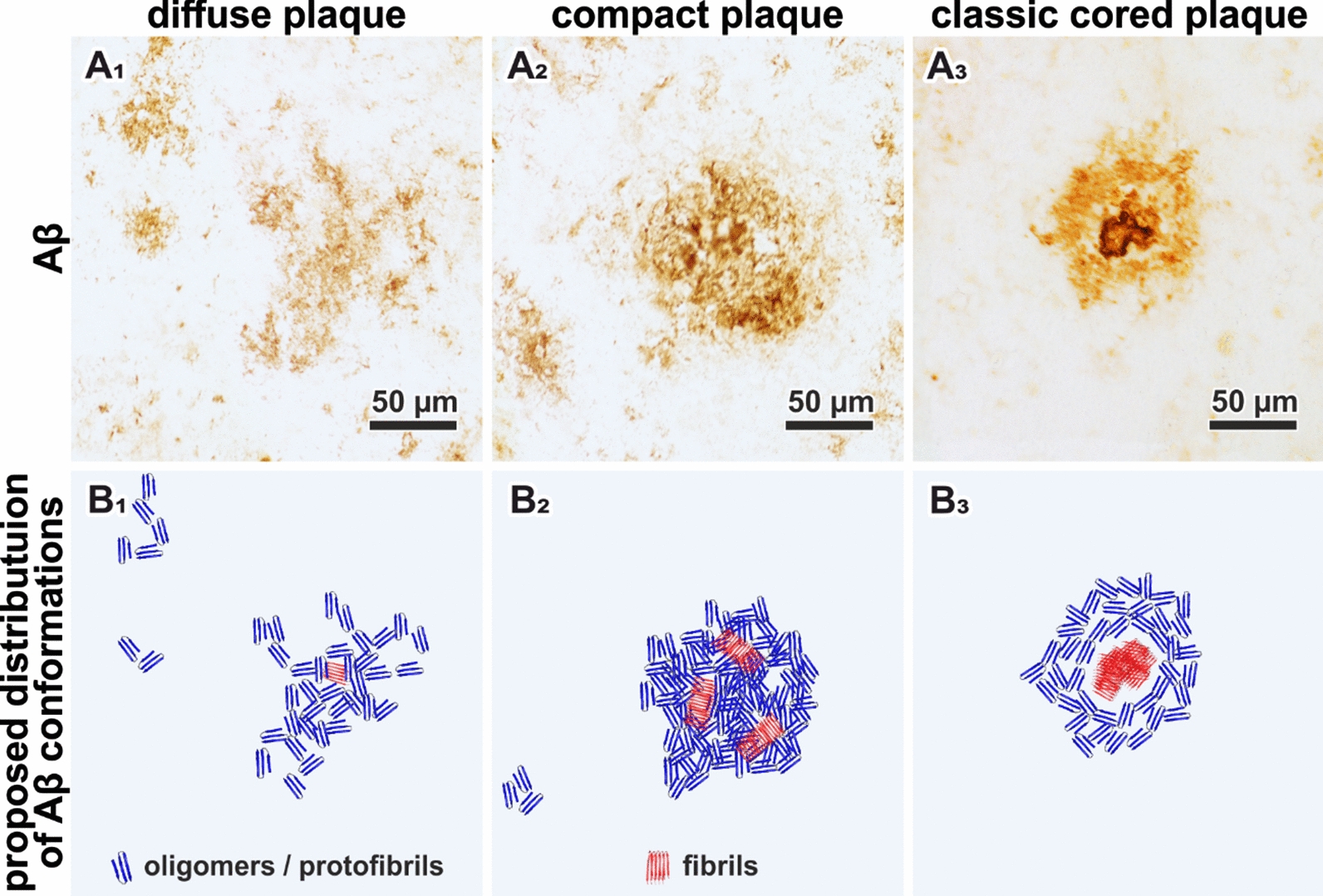
Proposal of Aβ conformations in the different plaque types. **A** depicts the exemplary plaques from Fig. 2. **B** Based on our observations, we propose the depicted composition of Aβ conformations in the different plaque types. The symbols are used to indicate the hypothetical location, density, and mixture of Aβ conformation in a simplified fashion

We observed protein clusters in diffuse plaques (Fig. 2B_2_), which might originate from parenchymal Aβ depositions or Aβ-enriched cells, which would align with descriptions of internalized Aβ prior to parenchymal infestation [13, 25, 33]. Diffuse plaques displayed increased β-sheet levels compared to control tissue (Fig. 5B). The main β-sheet band is symmetric around 1630 cm^−1^ (Fig. 4B). The marker band 1693 cm^−1^ for anti-parallel β-sheets is not notably decreased in difference spectra (Additional file 1: Fig. 5). Taken together, this indicates that the Aβ in diffuse plaques is primarily arranged in antiparallel β-sheets and parallel β-sheets with a low number of strands. We conclude that diffuse plaque contain mostly oligomeric and protofibrillar Aβ, yet in low concentrations (Fig. 6B_1_). This aligns well with the assumption that diffuse plaques are the starting point of plaque development [34].

Compact plaques usually displayed homogenously distributed β-sheet levels, while protein levels either appeared either homogenous or centrally localized (Fig. 2B_3_). Those centralized protein clusters were usually indistinguishable in Aβ-IHC and β-sheet images (Fig. 2A_3_, C_3_). FTIR imaging might be capable of detecting an emerging core in these plaques. However, the observed protein might also originate from proteins other than Aβ. Further investigations are necessary to clarify the origin of these observations. Furthermore, we observe a broad distribution of β-sheet levels in compact plaques (Fig. 5B). This aligns with reports, which state that not all compact plaques stain positive with Congo Red or Thioflavin [19, 20]. The Amide I band analysis indicates a significantly increased contribution of large parallel β-sheet structures in compact plaques (Figs. 4, 5). Thereby, we deduce that compact plaques contain an increased content of β-sheet Aβ, including varying amounts of Aβ fibrils (Fig. 6C_2_). Therefore, we propose that compact plaques are a heterogeneous group of intermediate states in plaque development, which cannot easily be differentiated with the most common anti-Aβ staining methods.

The cores of classic cored plaques display high levels of β-sheet Aβ (Fig. 2). The strong band at 1628 cm^−1^ and the decreased band around 1693 cm^−1^ imply high levels of large parallel β-sheet structures. From this, we conclude an abundance of Aβ fibrils in the cores (Fig. 6C_3_). This is consistent with amyloid-marker-based studies [2, 15, 65]. Nevertheless, our data suggests that plaque cores are not spheres of pure Aβ, as their usually dense appearance in Aβ-IHC images may suggest. FTIR measurements detect lipids within the cores (Fig. 3C), which aligns with a previous report [46]. This may imply (i) cellular involvement, for example of infiltrating microglia or astrocytes extensions [2, 14], or the presence of processes that (ii) actively or (iii) passively integrate membranous material in the cores. Neighboring neurites may also contribute to the lipid signal, as the spatial resolution of FTIR imaging is limited. Recently, evidence for lipids and membranous material in Lewy bodies in Parkinson’s disease has been reported [68]. Despite the ubiquitous abundance of lipids in the brain, this similarity of the two pathological phenomena is interesting, because it indicates cellular involvement in their respective developments; either as unintentional incorporation of e.g. cell fragments, or by cell-driven deposition [52]. Further research will be necessary to better understand the cellular involvement in the formation of Aβ plaques and other neuropathologies.

In contrast to the central plaque core, we observed low levels of β-sheets in the corona of classic cored plaques (Figs. 2, 3), indicating a low content of β-sheet Aβ in the corona. This appears to be the reason why we do not observe significant differences between compact plaques and classic cored plaques in the statistical analysis (Fig. 5). The statistical analysis is based on the mean spectra of plaques, which include the core and corona, in case of the classic cored plaques (Additional file 1: Fig. 4). Accordingly, the relatively large corona appears to compensate for the highly fibrillar core. This implies that the Aβ composition in the corona is similar to that in diffuse plaques; featuring low contents of mainly oligomeric and protofibrillar Aβ (Fig. 6C_3_). The substantial differences between cores and coronas may be due to the influence of inflammatory cells and the subsequent disaggregation and/or displacement of Aβ fibrils. Several studies have placed activated microglia in the focus of plaque core formation [2, 60, 61, 82]. We observe the characteristic gaps that glia cells leave in Aβ-IHC images of plaques (Fig. 2A), indicating glial involvement in the here investigated plaques. Microglia have been shown to dissolve Aβ fibrils into oligomers [36]. We suspect that such Aβ oligomers may be contained in the corona. Microglial involvement may also be a reason for the relatively low protein levels, which we observe in coronas (Fig. 2B_4_), as the infiltrating microglia might displace protein with their lipid-rich cell bodies [2, 58, 82]. Additionally, several studies have found neurites and various dystrophic cell fragments in the corona [17, 19], which presumably contribute to Aβ displacement.

Raman data of identical tissue areas validate FTIR results on classic cored plaques and reproduces the distribution of β-sheet protein in a side-by-side comparison to FTIR (Additional file 1: Fig. 6). As similar results were obtained with an independent technique, our findings are verified, confirming the reliability of our label-free approach.

In conclusion, our novel approach allowed us to track the progression of Aβ fibrillation alongside the plaque development sequence, for the first time label-free, in human brain tissue thin sections. Our observations of successive accumulation and fibrillation of β-sheet structured Aβ gives implications for therapeutic approaches and supports that the proposed plaque-type sequence (diffuse, compact, classic cored plaques) describes the development stages of Aβ plaques in AD.

## Supplementary information

**Additional file 1.** Supplementary Information. Materials and Methods - Raman. **Table S1**. Case details. **Table S2**. Nomenclature. **Fig. S1**. Investigation of spectral alterations during long measurements. **Fig. S2**. Further exemplary classic cored plaque. **Fig. S3**. Mie correction. **Fig. S4**. Plaque masks. **Fig. S5**. Difference spectroscopy. **Fig. S6**. Validation with Raman.

## Abbreviations

AD: Alzheimer’s disease
NFT: neurofibrillary tangle
Aβ: amyloid beta
FTIR: Fourier-transform infrared
APP: amyloid precursor protein
Aβ-IHC: anti-Aβ immunohistochemistry
LPS: superior parietal lobule
FOV: field of view
ROI: region of interest
EMSC: extended multiplicative signal correction
HCA: hierarchical cluster analysis
