# Supplementary material for: Label-free vibrational imaging of different Aβ plaque types in Alzheimer’s disease reveals sequential events in plaque development
Source: Acta Neuropathol Commun. 2020 Dec 11;8:222. doi: 10.1186/s40478-020-01091-5 (PMC7733282; doi:10.1186/s40478-020-01091-5)
Supplement: Supplementary file 1 — Additional file 1. Supplementary Information. Materials and Methods - Raman. Table S1. Case details. Table S2. Nomenclature. Fig. S1. Investigation of spectral alterations during long measurements. Fig. S2. Further exemplary classic cored plaque. Fig. S3. Mie correction. Fig. S4. Plaque masks. Fig. S5. Difference spectroscopy. Fig. S6. Validation with Raman. [file 40478_2020_1091_MOESM1_ESM.docx]

Supplementary Information for

**Label-free vibrational imaging of different Aβ plaque types in Alzheimer’s disease reveals sequential events in plaque development**

Dominik Röhr^1,2^, Baayla D.C. Boon^3,4^, Martin Schuler^1,2^, Kristin Kremer^1,2^, Jeroen J.M. Hoozemans^3^, Femke H. Bouwman^4^, Samir F. El-Mashtoly^1,2^, Andreas Nabers^1,2^, Frederik Großerueschkamp^1,2^, Annemieke J.M. Rozemuller^3^, Klaus Gerwert^1,2^

^1^ Ruhr University Bochum, Center for Protein Diagnostics (ProDi), Biospectroscopy, Germany

^2^ Ruhr University Bochum, Faculty of Biology and Biotechnology, Department of Biophysics, Germany

^3^ Amsterdam UMC - location VUmc, Department of Pathology, Amsterdam Neuroscience, Amsterdam, The Netherlands

^4^ Amsterdam UMC - location VUmc, Department of Neurology, Amsterdam Neuroscience, Alzheimer Center Amsterdam, The Netherlands

**Email:**  [klaus.gerwert@rub.de](mailto:klaus.gerwert@rub.de)

**This file includes:**

Material and methods - Raman

Supplementary Tables 1 to 2

Supplementary Figures 1 to 6

References**Materials and Methods – Raman**

**Raman microspectroscopy.** Raman spectral imaging was performed using the WITec alpha300 RA confocal Raman microscope (WITec, Ulm, Germany) as described before [1, 10]. A single-frequency diode laser of 785 nm (Toptica Photonics AG, Munich, Germany) was utilized for excitation with an output power of approximately 300 mW. The laser beam was collimated with an achromatic lens, passed through a holographic band-pass filter and subsequently focused on the sample with a Nikon S Plan Fluor (20x/0.45) dry-objective. The sample was placed on a piezoelectrically driven scanning stage and the back-scattered light was collected with the same objective and passed through the holographic edge filter into a multimode fiber (d = 50 µm). A 300 mm focal length monochromator with a 600/mm grating, blazed at 750 nm was utilized and the Raman spectra were detected by a back-illuminated deep‑depletion charge-coupled device (-60°C). Dry air was continuously purged onto the sample to avoid sample degradation. Raman spectra were acquired with a pixel resolution of 1 µm and 1 second integration time per pixel. Due to the small area covered by Raman and difficulties to localize plaques in unstained tissue, even when guided by adjacent Aβ-IHC stained sections, only two classic cored plaques were measured. Given the restricted plaque quantity, Raman data was used for validation of FTIR, rather than providing new results.

**Raman spectral data preparation and data analysis.** All spectra were smoothed by Savitzky-Golay algorithm, baseline-corrected using a fourth-order polynomial with a noise threshold of 3 and a custom spectral fit mask with the Project FOUR software (WITec, Ulm, Germany). Raman spectra of the tissue regions that displayed strong (auto-)fluorescence or noise were excluded from data analysis. Cosmic peak correction was done by replacing peaks with neighboring values in the spectra. Peak ratios were calculated to visualize the ratio between protein and lipids, as well as the degree of protein misfolding, analog to FTIR analysis (supplementary Tab. 2). We used the ratio I_1665_ /I_1654_ of the Raman-active sub-bands of the Amide I band to evaluate the degree of protein misfolding in Raman, analog to the A_1630_/A_1655_ ratio in FTIR. The ratio I_1659_ /I_CH Total_ compares the total Amide I band of the protein back bone and the CH_2_ & CH_3_ deformation bands, mostly of fatty acids and protein side chains. The selected bands represent the same molecular classes in Raman and FTIR, namely protein for the Amide bands, and a sum of lipid and protein for the CH_2_ & CH_3_ groups.**Tables**

**Tab. 1. Case details.** Abbreviations: AD Alzheimer’s disease; NA not applicable; PMI post-mortem interval; # number of; † death; ^a^ no plaques but only dystrophic angiopathy in gallyas staining; ^b^ severe tauopathy related to vascular amyloid, not qualifying Braak staging.

| Case # | Group | Gender | Onset age | Age † | Cause of † | PMI hh:mm | ABC (Montine et al.) | # diffuse plaques | # compact plaques | # classic cored plaques |
| --- | --- | --- | --- | --- | --- | --- | --- | --- | --- | --- |
| 1 | Control | Male | NA | 83 | Myocard infarct | 5:15 | A1 B1 C0 | NA | NA | NA |
| 2 | Control | Female | NA | 92 | Heart failure | 6:35 | A0 B2 C0 | NA | NA | NA |
| 3 | Control | Female | NA | 78 | Euthanasia | 7:10 | A1 B1 C0 | NA | NA | NA |
| 4 | AD | Male | 60 | 65 | Cardiac insufficiency | 8:50 | A3 B3 C3 | 0 | 1 | 1 |
| 5 | AD vascular variant | Male | 77 | 80 | Dehydration | 5:05 | A3 B3 C0^a^ | 3 | 9 | 7 |
| 6 | AD | Female | 75 | 84 | Cardiac arrest after cachexia | 6:30 | A3 B3 C3 | 8 | 5 | 16 |
| 7 | AD | Female | 64 | 76 | Unclear | 10:45 | A3 B3 C3 | 45 | 6 | 4 |
| 8 | AD | Male | 48 | 59 | Dehydration / cachexia | 7:35 | A3 B3 C3 | 0 | 6 | 4 |
| 9 | AD | Male | 59 | 65 | Pneumonia | 3:32 | A3 B3 C3 | 4 | 1 | 26 |
| 10 | AD vascular variant | Female | 75 | 78 | Dehydration / cachexia | 4:20 | A3 NA^b^ C0^a^ | 8 | 4 | 2 |

**Tab 2. Nomenclature of mean values.** The mean absorbance *A* or mean Raman intensity *I* was calculated within the respective peak range. A linear baseline correction was applied to FTIR spectra within the ranges stated below.

| Nomenclature | Observed vibration | Peak range | Baseline range |
| --- | --- | --- | --- |
| A_1545_ | Amide II [4] | 1535 - 1555 | 1345 - 1780 |
| A_1630_ | Amide I of β-sheets [2] | 1630 | - |
| A_1655_ | Amide I of non-β-sheets | 1655 | - |
| A_Total CH_ | CH_2_ and CH_3_ stretching | 2800 - 2995 | 2750 - 3100 |
| I_1659_ | Amide I total | 1650 - 1673 | - |
| I_1654_ | Amide I of non-β-sheets | 1644 - 1659 | - |
| I_1665_ | Amide I of β-sheets | 1664 - 1679 | - |
| I_Total CH_ | CH_2_ and CH_3_ deformation | 1430 - 1446 | - |

**Figures**

**Investigation of spectral alterations in native brain samples during long measurements:**

We carefully prevented chemical alterations of our samples to approach *in vivo* conditions as close as possible. Nonetheless, the samples were kept in dried air during defrosting and spectral measurements, which usually lasted for several days. As we analyze minute chemical changes, some of which associated to oxidation processes, the ambient oxygen constitutes a potentially disruptive factor.

In order to characterize possible spectral changes that originate from prolonged exposure to dry air, we conducted a simple experiment. We measured a sample with FTIR, as described above, and instead of staining right away, we kept the sample in a closed, dry-air-purged container for about 3 weeks, repeated the FTIR measurement afterwards and then stained subsequently. We extracted plaque spectra from 10 plaques, contained in both measurements to conduct a precise comparison.

The mean plaque spectra are presented in supplementary Fig. 1. After prolonged dry air exposure, we observe the emergence of bands around 1322 cm^-1^, 1160 cm^-1^ and 1095 cm^-1^. The assignment of these bands is difficult, because of the multitude of overlapping bands in the fingerprint region. From literature we deduce, that a possible origin of the bands might be the stretching vibrations of C-O-R groups, whereas R represents a manifold of possible extensions [8]. Fortunately, the spectral ranges we used for the analysis of plaques do not show alterations after a prolonged exposure to dry air.

**Fig. 1.**
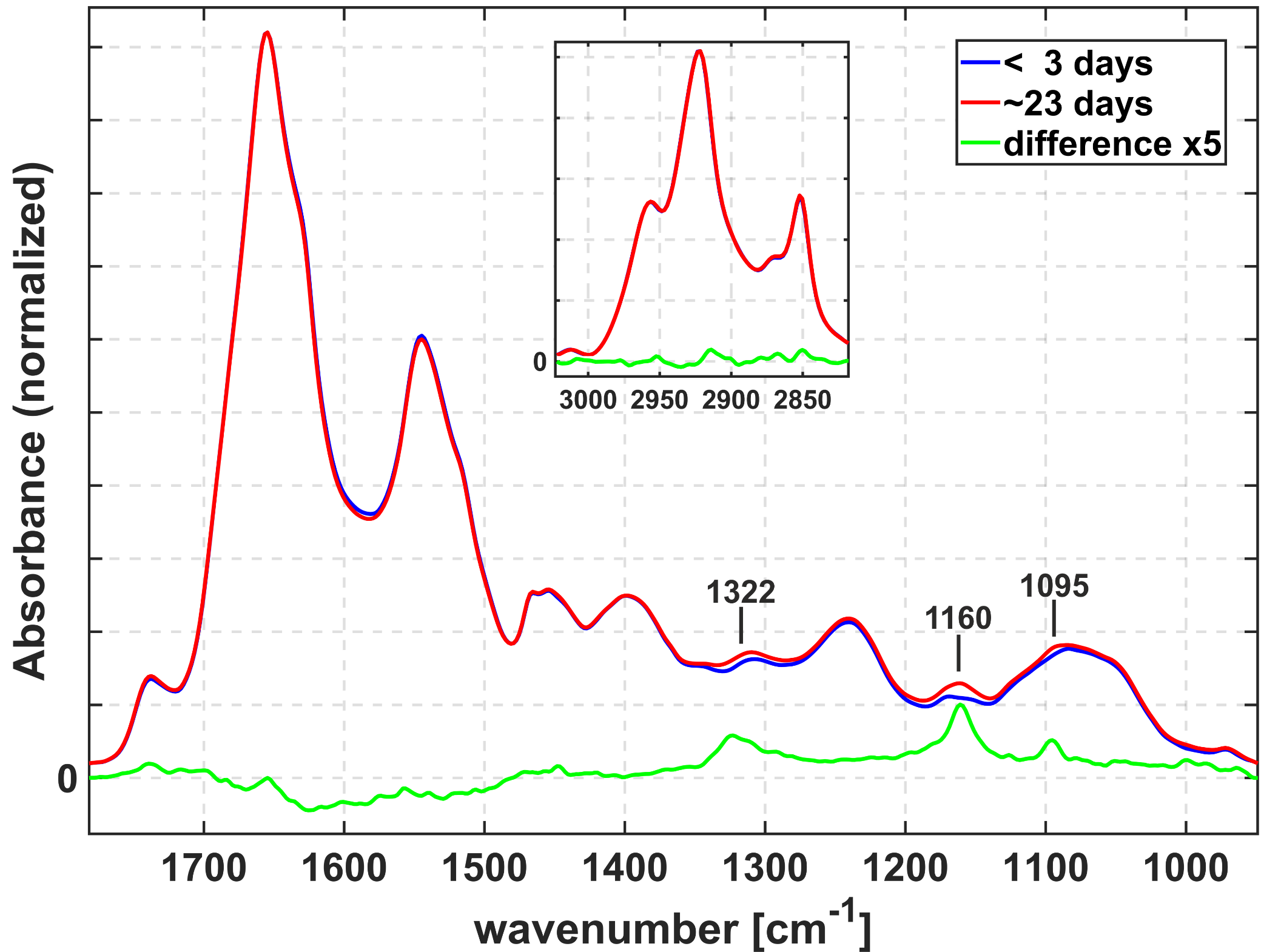
**Sample ageing.** Area-normalized mean plaque spectra from FTIR measurements shortly after defrosting (blue) and about three weeks later (red). The 5-fold difference of both spectra is plotted in green.
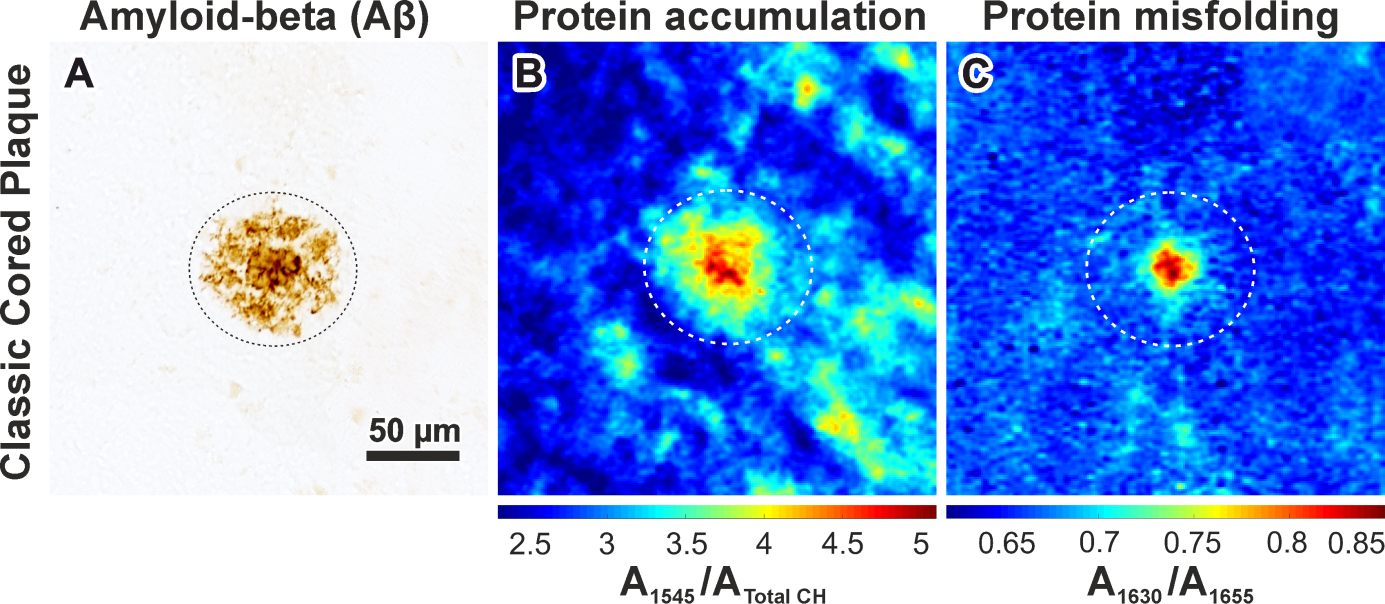
**Fig. 2. Further exemplary classic cored plaque.** Analog to Fig. 2.**
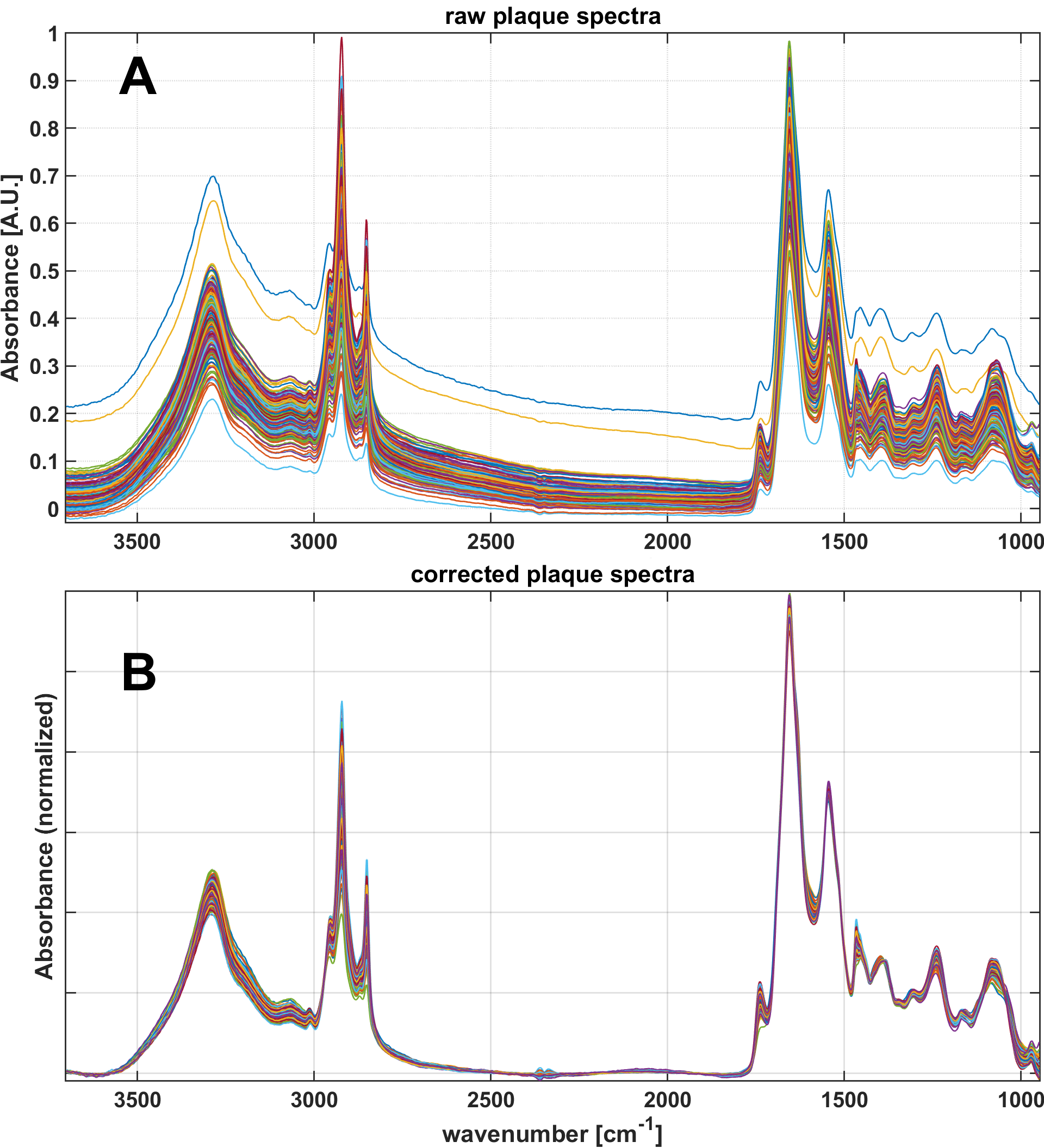
Fig. 3. Mie Correction.** Typical spectra before and after application of Mie scattering correction based on extended multiplicative signal correction (EMSC) [9].**Fig. 4. Plaque masks. A** Original Aβ-IHC staining. **B** Overlay of staining image and masks. **C** Masks
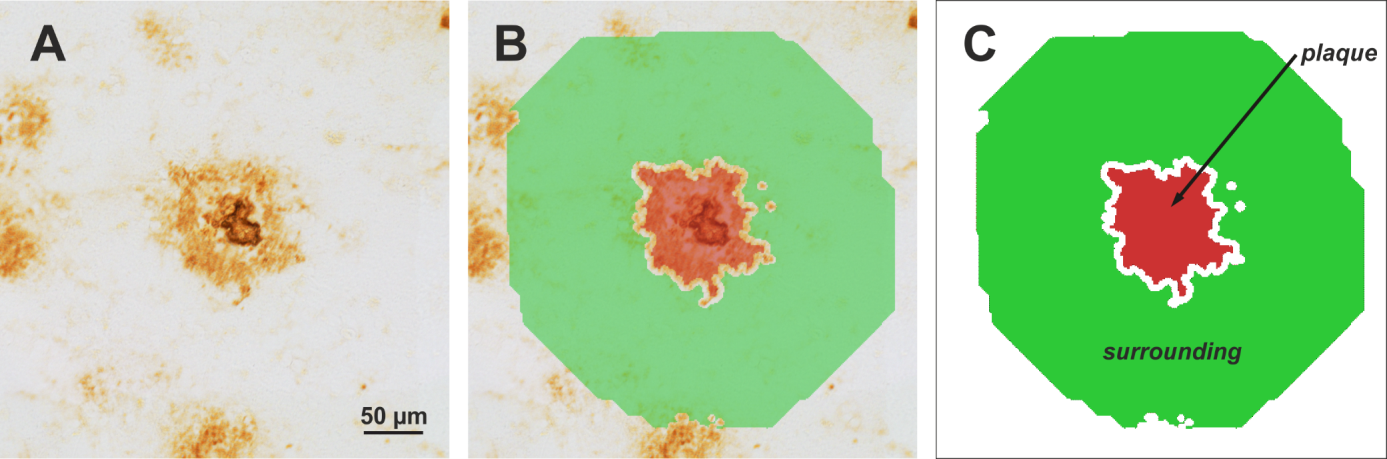
 for the plaque area (red) and the surrounding area (green)**
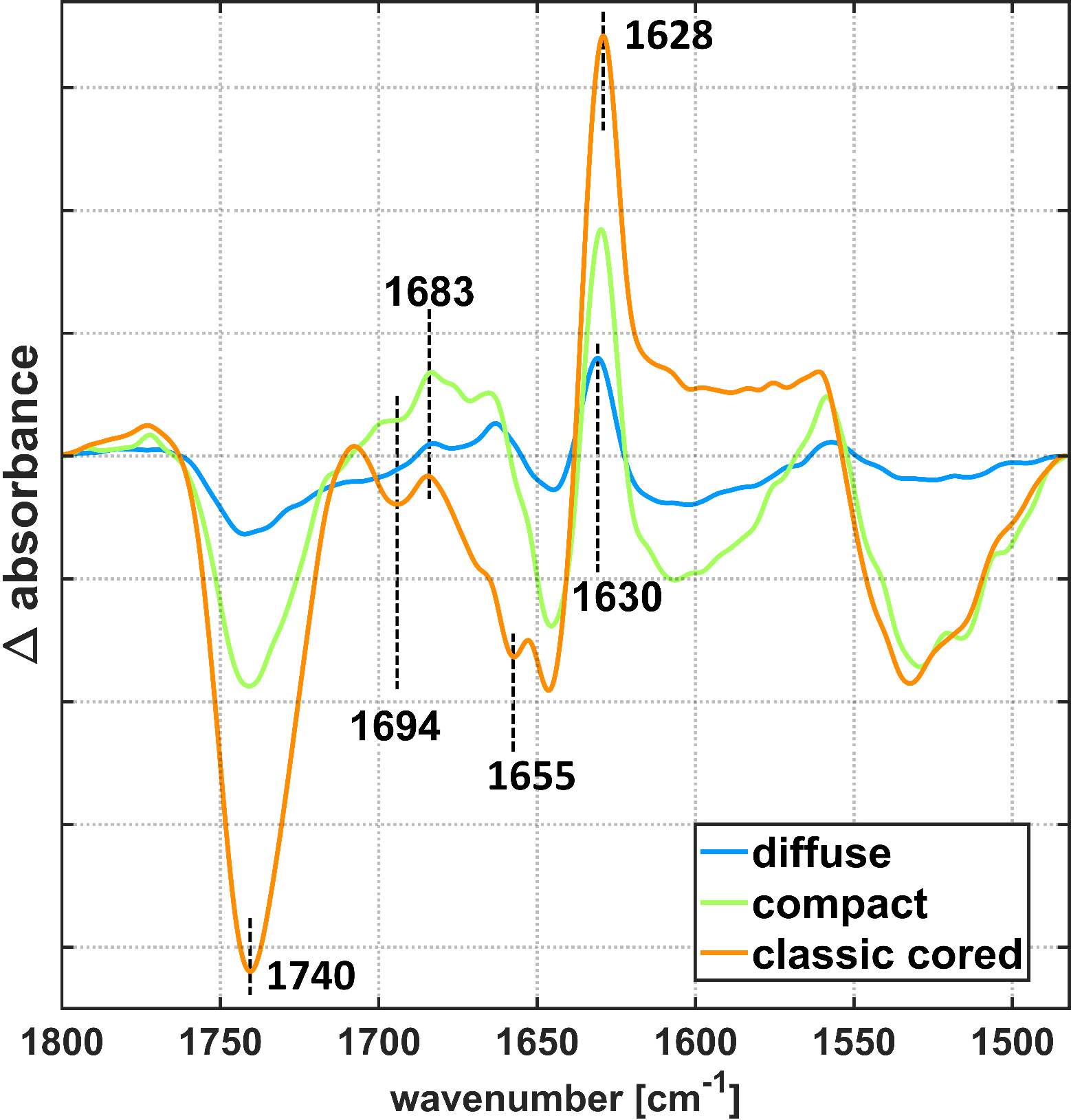
Fig. 5. Difference spectroscopy.** Difference spectra between mean *plaque spectra* and their corresponding *surrounding spectra*. Relevant bands are marked.

Around 1694 cm^-1^, we observe (i) no clear band in diffuse plaques, (ii) a dip in compact plaques, and (iii) a clear local minimum in classic cored plaques. All plaque types display local maxima around 1683 cm^-1^ and 1630 cm^-1^.**Validation with Raman**

In the Raman measurements (supplementary Fig. 6), we observed auto-fluorescence in small parts of the tissue that might originate from lipofuscin deposits [5]. However, Raman data can still be used to confirm the findings of FTIR. One distinction between FTIR and Raman concerns the Amide I band. The respective bands of β-sheets are most prominent around 1666 cm^‑1^ in Raman and around 1630 cm^‑1^ in FTIR [6]. The observed Amide I band shift in the plaque of human tissue in the present Raman study (supplementary Fig. 6A_3_) is consistent with that monitored by stimulated Raman scattering using fresh frozen AD mouse brain sections [3]. Furthermore, a major difference between FTIR and Raman is the confocality as well as higher spatial resolution provided by Raman imaging. These differences are visible in the integrated absorption signal of all tissue layers is shown for the FTIR images (supplementary Figs. 6B_1_ and 6C_1_), but a z-layer specific emission intensity is displayed in the Raman images (supplementary Figs. 6B_2_ and 6C_2_), resulting in an altered appearance of the plaque in Raman images. This includes the formation of β-sheet rich structures, most likely Aβ fibrils, in classic cored plaques.

**
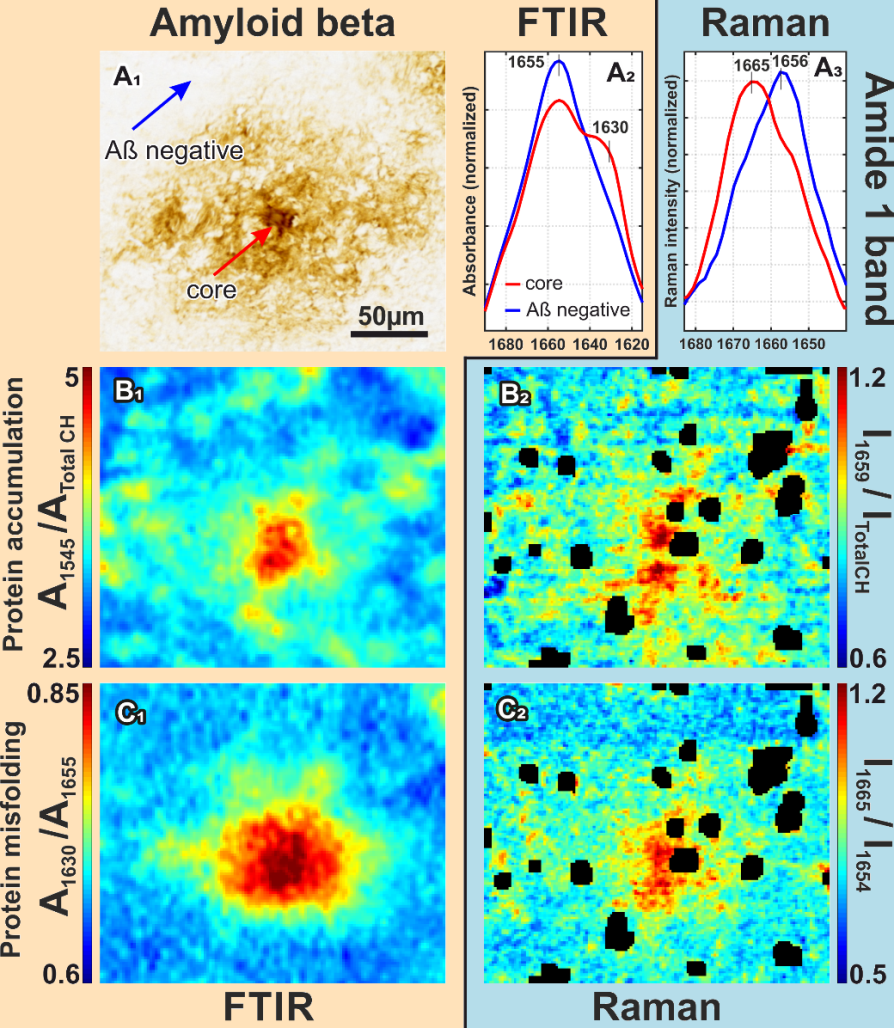
Fig. 6. Raman** spectral analysis of a classic cored plaque in direct comparison to FTIR. **A_1_** anti-Aβ staining of the plaque. The red and blue arrows indicate the regions corresponding to the spectra in A_2_ and A_3_. **A_2_** FTIR Amide I bands of the plaque core (red) and the Aβ negative region (blue). **A_3_** Raman Amide I bands of the plaque core (red) and the Aβ negative region (blue). All bands were extracted from corrected spectra and normalized on the same area under the line within the displayed ranges. **B_1_ and C_1_** display the protein accumulation and misfolding within the tissue, determined by FTIR, analog to Figs. 2B and 2C. **B_2_** **and C_2_** show the protein accumulation and misfolding within the plaque as determined by Raman. The blackened areas indicate pixel spectra, which were unsuitable for analysis due to strong fluorescence artefacts.**References**

1. Aljakouch K, Lechtonen T, Yosef HK, Hammoud MK, Alsaidi W, Kötting C, et al. (2018) Raman Microspectroscopic Evidence for the Metabolism of a Tyrosine Kinase Inhibitor, Neratinib, in Cancer Cells. Angew Chemie - Int Ed 57:7250–7254. doi: 10.1002/anie.201803394

2. Goormaghtigh E, Ruysschaert J-M, Raussens V (2006) Evaluation of the Information Content in Infrared Spectra for Protein Secondary Structure Determination. Biophys J 90:2946–2957. doi: 10.1529/biophysj.105.072017

3. Ji M, Arbel M, Zhang L, Freudiger CW, Hou SS, Lin D, et al. (2018) Label-free imaging of amyloid plaques in Alzheimer’s disease with stimulated raman scattering microscopy. Sci Adv 4:1–9. doi: 10.1126/sciadv.aat7715

4. Leskovjan AC, Lanzirotti A, Miller LM (2009) Amyloid plaques in PSAPP mice bind less metal than plaques in human Alzheimer’s disease. Neuroimage 47:1215–1220. doi: 10.1016/j.neuroimage.2009.05.063

5. Lochocki B, Morrema THJ, Ariese F, Hoozemans JJM, de Boer JF (2020) The search for a unique Raman signature of amyloid-beta plaques in human brain tissue from Alzheimer’s disease patients. Analyst 145:1724–1736. doi: 10.1039/C9AN02087J

6. Miyazawa T, Blout ER (1961) The Infrared Spectra of Polypeptides in Various Conformations: Amide I and II Bands. J Am Chem Soc 83:712–719. doi: 10.1021/ja01464a042

7. Sarroukh R, Cerf E, Derclaye S, Dufrêne YF, Goormaghtigh E, Ruysschaert JM, et al. (2011) Transformation of amyloid β(1-40) oligomers into fibrils is characterized by a major change in secondary structure. Cell Mol Life Sci 68:1429–1438. doi: 10.1007/s00018-010-0529-x

8. Socrates G (2001) Infrared and Raman Characteristic Group Frequencies: Tables and Charts, 3rd ed. J.Wiley and Sons LTD, Chichester

9. Solheim JH, Gunko E, Petersen D, Großerüschkamp F, Gerwert K, Kohler A (2019) An open‐source code for Mie extinction extended multiplicative signal correction for infrared microscopy spectra of cells and tissues. J Biophotonics 12. doi: 10.1002/jbio.201800415

10. Yosef HK, Frick T, Hammoud MK, Maghnouj A, Hahn S, Gerwert K, et al. (2018) Exploring the efficacy and cellular uptake of sorafenib in colon cancer cells by Raman micro-spectroscopy. Analyst 143:6069–6078. doi: 10.1039/c8an02029a
